# Changes in PD-1 expression on T lymphocyte subsets and related immune indicators before and after definitive chemoradiotherapy for esophageal squamous cell carcinoma

**DOI:** 10.1080/07853890.2024.2445190

**Published:** 2024-12-23

**Authors:** Xueling Shi, Hongyu Zhao, Jiaqi Yu, Peng Cai, Shixiang Zhou, Ning Yang, Duojie Li

**Affiliations:** a Department of Radiotherapy, The First Affiliated Hospital of Bengbu Medical University, Bengbu, Anhui, China; b Anhui Province Key Laboratory of Cancer Translational Medicine, Bengbu Medical University, Bengbu, Anhui, China

**Keywords:** PD-1, T lymphocyte subsets, NK cells, immunotherapy, chemoradiotherapy, esophageal squamous cell carcinoma

## Abstract

**Objective:**

This study aimed to observe the dynamic changes in the expression of T lymphocytes, natural killer (NK) cells, and PD-1 in patients with first-diagnosed esophageal squamous cell carcinoma (ESCC) before and after chemoradiotherapy (CRT) and evaluate the impact of PD-1 expression in peripheral blood on the short-term outcome of patients with ESCC.

**Patients and Methods:**

Seventy-three patients with ESCC who were treated with definitive CRT were enrolled. Before and after CRT, flow cytometry was used to detect thePD-1 expression in the peripheral blood and related immune indicators. Peripheral blood from 10 healthy individuals was used as control.

**Results:**

The percentages of CD3^+^ (*p* = 0.018), CD4^+^ (*p* < 0.001), and CD8^+^ T cells (*p* < 0.001); NK cells (*p* = 0.009); and the CD4^+^/CD8^+^ ratio (*p* < 0.001), as well as PD-1^+^CD3^+^ (*p* < 0.001), PD-1^+^CD4^+^ (*p* < 0.001), and PD-1^+^CD8^+^ (*p* < 0.001) T cells, before CRT significantly differed from those in the post-CRT group. The percentages of PD-1^+^CD8^+^ T cells differed significantly between the radiotherapy alone and CRT groups (*p* < 0.05). PD-1 expression in CD3^+^, CD4^+^, and CD8^+^ T cells significantly decreased in patients achieving overall response rate (all *p* < 0.05). Compared with those in the incomplete response group, PD-1^+^CD8^+^ T cells significantly decreased in the CR group (*p* < 0.05).

**Conclusion:**

CRT aggravated immunosuppression and increased PD-1 expression in T lymphocyte subsets in patients with ESCC, possibly related to the radiation field. PD-1 expression in T lymphocyte subsets can predict short-term outcomes in patients and provide a theoretical basis for the sequential application of PD-1 immunosuppressants after radiotherapy and chemotherapy.

## KEY MESSAGES

1. Chemoradiotherapy (CRT) reduced the overall immune function of patients with esophageal squamous cell carcinoma (ESCC), and the degree of decline was related to the irradiation area.
2. PD-1 expression in T lymphocytes increased considerably after CRT, suggesting that the sequential application of PD-1 immunosuppressants after radiotherapy and chemotherapy can be beneficial.
3. PD-1 expression on T lymphocytes may serve as a potential indicator for predicting the short-term outcomes of ESCC.

## 1. Introduction

Esophageal Cancer (EC) is a malignant tumor that poses a serious threat to human health. According to the global cancer data released by the International Agency for Research on Cancer (IARC) in 2020, EC ranks 7th in terms of incidence and 6th in terms of mortality worldwide [1]. More than half of the new cases and deaths worldwide occur in China [2]. The histological subtypes of EC vary among countries. Adenocarcinoma is the predominant cancer type in European and American countries, whereas esophageal squamous cell carcinoma (ESCC) accounts for over 95% of cases in China [3]. Surgical intervention is the optimal treatment of choice for patients with early-stage EC. However, owing to the highly malignant nature and rapid progression of the disease, many patients are diagnosed at an advanced stage, missing the window for surgery. Therefore, 50–60% of patients with EC are not candidates for curative resection. For many of these patients, guidelines recommend definitive chemoradiotherapy (CRT) as the standard treatment option [4]. If a patient is intolerant to CRT, radiotherapy alone is the primary alternative. However, radiotherapy alone was associated with a shorter median overall survival compared to CRT [5]. New combination therapies are urgently needed to improve patient outcomes. Therefore, radiotherapy or CRT combined with immunotherapy is receiving increasing attention.

Tumor progression is usually accompanied by low immune function, and the host immune system plays an important role in the efficacy of CRT [6]. We found that CRT affects the differentiation pathways and functions of T cells in the tumor microenvironment [7], Long-term weakening of T cell immune function after CRT can change the host immune response and may be a key factor affecting the prognosis and outcome of EC [8]. Immunotherapy has become a research hotspot, and many clinical trials are ongoing to evaluate the effectiveness of combining immune checkpoint inhibitors with radiotherapy and chemotherapy [9]. Immune checkpoint inhibitors achieve antitumor effects by blocking negative mechanisms in the immune system [10]. PD-1 acts as an inhibitory receptor that hinders T-cell activation by interacting with specific ligands. In contrast, PD-1 inhibitors prevent PD-1 from binding to PD-L1, thus restoring immune surveillance and triggering T-cell antitumor responses [11]. Abnormal expression of the PD-1/PD-L1 signaling pathway has been linked to tumor progression, metastasis, and prognosis in various malignant tumors [12]. This study aimed to investigate the correlation between peripheral blood lymphocyte subsets, natural killer (NK) cells, PD-1 expression, clinicopathological characteristics, and short-term outcomes and to evaluate the clinical application value of PD-1 expression in the use of immunosuppressants.

## 2. Patients and methods

### 2.1. Patients

This prospective study involved 73 untreated patients with esophageal squamous cell carcinoma (ESCC) who underwent definitive CRT at the First Affiliated Hospital of Bengbu Medical University between September 2022 and April 2024. Inclusion criteria comprised: (1) Age 18 or above, (2) first-time diagnosis without prior radiotherapy/chemotherapy/immunotherapy, (3) newly diagnosed ESCC confirmed by histopathology, (4) Karnofsky performance status ≥70 points, and (5) absence of previous malignant tumors or serious medical conditions. Of the 73 patients, 44 were male (60.27%) and 29 were female (39.73%), and the average age was 76.42 ± 8.35 years. There were 22 cases of upper EC, 33 of middle EC, and 18 of lower EC. Fifty-three cases presented with lesions longer than 5 cm. Among these patients, 19.18% had M1 stage disease, and all patients with M1 stage disease without distant organ metastasis had supraclavicular lymph node metastasis. Most patients (61.64%) received definitive CRT. Additional baseline characteristics are summarized in Table S1.

The study protocol was approved by the Ethics Committee of the First Affiliated Hospital of Bengbu Medical College (Ethics Committee Number: 2021KY032). All patients signed informed consent forms for radiotherapy, and healthy volunteers in the control group signed consent forms for blood sample testing. Before radiotherapy and after 60 Gy radiotherapy, peripheral blood immune indicators and PD-1 expression were detected. Blood samples from 10 healthy volunteers were randomly selected from the staff of our hospital as controls, including 6 males and 4 females aged 60–80 years, with a median age of 72 years.

### 2.2. Treatment

All patients received three-dimensional conformal radiotherapy or intensity-modulated radiotherapy. Definitive radiotherapy (*n* = 30, 41.10%) or definitive concurrent CRT (*n* = 43, 58.90%) was administered based on the patient’s clinical stage and physical condition. Radiotherapy plans were generated using Pinnacle 11.0 or Monaco 5.11.03. The radiation dose was 60 Gy, 2.0 Gy each time, once daily, 5 times per week. The plan was standardized to 95% of the planned tumor volume, receiving 100% of the prescribed dose. All the organs at risk (OAR) are controlled to be below the safe range. The target volume for radiotherapy was the planning target volume (PTV) generated in the radiotherapy plan. The gross tumor volume (GTV) included the primary tumor of EC. The metastatic lymph node gross tumor volume (GTVnd) only included metastatic lymph nodes with the shortest diameter of > 1 cm or lymph nodes of the tracheoesophageal groove with the shortest diameter of > 5 mm. The clinical target volume (CTV) included subclinical lesions (lesion area expanded by 3 cm along the longitudinal axis of the esophagus) and involved lymphatic drainage areas. The PTV was determined by magnifying the CTV by 0.5 cm in each direction, and our hospital’s treatment planning system was used to automatically calculate the PTV. The results showed that the PTV radiotherapy target area ranges from 106.20 to 892.60 cm^3^, and the median target area was 262.80 cm^3^. And the GTV radiotherapy target volume ranges from 10.80-129.00 cm3, and the median target volume is 31.22 cm^3^. Chemotherapy regimens typically consist of two main types: platinum-based intravenous chemotherapy and oral S-1 chemotherapy. Concurrent chemotherapy often involves one or two weekly cycles of intravenous cisplatin or nedaplatin (75 mg/m^2^) starting on the first day of radiotherapy. concurrent S-1 was administered orally twice daily on radiotherapy days (total daily dosage, 40-60 mg/m2 based on body surface area). S-1 was administered orally on days 1 to 14 of radiotherapy and repeated every three weeks. In cases in which patients are unable to tolerate concurrent CRT, the dosage of chemotherapy drugs can be reduced or suspended to ensure the completion of radiotherapy.

### 2.3. Sample collection

#### 2.3.1. Before treatment

Blood was collected from all patient on an empty stomach early in the morning after diagnosis. Venous blood samples were collected in EDTA tubes, marked, numbered, and immediately stored in a 4 °C environment. Flow cytometry was performed within 6 h.

#### 2.3.2. End of radiotherapy

Fasting blood was collected from all patients in the early morning near the after 60 Gy radiotherapy. Venous blood samples were collected in EDTA tubes, marked, numbered, and immediately stored in a 4 °C environment for flow cytometry detection within 6 h.

#### 2.3.3. Healthy control group

The control group consisted of 10 healthy participants of similar sex and age who underwent physical examinations at the physical examination center of our hospital during the same period. Blood was also collected on an empty stomach in the early morning, and venous blood samples were collected in EDTA tubes, marked and numbered, and immediately placed in a 4 °C environment for flow cytometry detection within 6 h.

### 2.4. Main reagents and instruments

Phosphate buffer saline (PBS) was purchased from Gibco in the United States; CD279 BV421 (catalog number: 562516, clone number: TH12.1) and 6-color TBNK (CD3 FITC/CD16 PE+CD56 PE/CD45 PE-Cy5/CD4 PE-Cy7/CD19 APC/CD8 APC-Cy7) were purchased from Jiangsu Tian hang Medical Technology Co., Ltd.; red blood cell lysate (BD FACS Lysing Solution) was purchased from BD Bioscience, USA; the high-speed centrifuge was purchased from Anhui Zhongkezhongjia Scientific Instrument Co. Ltd.; and the flow cytometer was purchased from BD Bioscience, USA. A −80 °C low temperature refrigerator was purchased from San YO, Japan, and a pipette gun was purchased from Eppendorf, Germany.

### 2.5. Experimental steps

Two EDTA anticoagulation tubes were labeled T1 and T2. In T1, 100 μL of peripheral blood was added along with the following flow cytometry antibodies: 10 μL 6-color and 2 μL CD279-BV421 and incubated for 30 min at 4 °C in the dark. Subsequently, the cells were lysed by the BD FACS Lysing Solution for 15 min at 4 °C and washed twice with cold PBS. The corresponding isotype antibody was added to the T2 tube, mixed thoroughly, and incubated at room temperature in the dark for 30 min. This was made to set compensation and gates. Specimen assays were performed by flow cytometry.

### 2.6. Data collection

**Figure 1.**
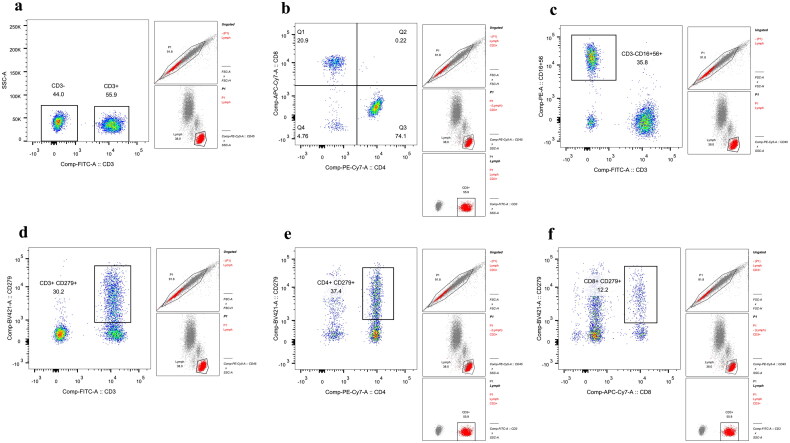
Flow chart of T lymphocyte subsets, PD-1, and NK cells. (a) The box on the right side represents the proportion of CD3^+^ cells, that is, T lymphocytes. (b) The box in Q1 represents the proportion of CD3^+^CD8^+^ cells, that is, CD8^+^ T cells, whereas the box in Q3 represents the proportion of CD3^+^CD4^+^ cells, that is, CD4^+^ T cells. (c) The circle in the box represents the proportion of CD3^-^CD16^+^CD56^+^ cells, that is, NK cells. (d) The circle in the box represents the proportion of CD3^+^CD279^+^ cells. The proportion represents that of PD-1 expression among the total T cells. (e) The proportion of CD4^+^CD279^+^ cells circled in the box represents that of PD-1 expression in CD4^+^ T cells. (f) The circle in the box represents the proportion of CD8^+^CD279^+^ cells, representing the proportion of PD-1 expression among CD8^+^ T cells.

PBS (350 μL) was added to both tubes, and flow cytometry was applied for detection and data collection. Before detection, the quality control microspheres of the flow cytometer were used for calibration, and the sample was loaded. The test template must set the gates on a scatter plot to circle the lymphocyte population. FMO control, namely T2 tubes, and single fluorescent tubes, were used to adjust the voltage and fluorescence compensation. We collected 20,000 cells from each sample and analyzed T lymphocyte subpopulations, percentage, and cell number. The subsequent specimen operation steps were the same as those described prior, and all blood samples were prepared and measured within 6 h of collection. The gating scheme is shown in Figure 1. The proportion of CD3^+^ T cells was detected by setting the gate with lymphocytes to obtain the proportion of CD3^+^ T cells. The proportion of CD4^+^ T cells was detected by setting the gate with CD3^+^ T cells to obtain the proportion of CD3^+^CD4^+^ cells. The proportion of CD8^+^ T cells was determined by setting the gate with CD3^+^ T cells to obtain the proportion of CD3^+^CD8^+^cells. The proportion of NK cells was detected by setting the gate with CD3^-^ T cells to obtain the proportion of CD3^-^CD16^+^CD56^+^ cells. The proportion of PD-1^+^CD3^+^ cells was detected by setting the gate with lymphocytes to obtain the proportion of CD3^+^CD279^+^ cells. The proportion of PD-1^+^CD4^+^cells was detected by setting the gate with CD3^+^ to obtain the proportion of CD4^+^CD279^+^ cells. The proportion of PD-1^+^CD8^+^ cells was detected by setting the gate with CD3^+^ to obtain the proportion of CD8^+^CD279^+^ cells.

### 2.7. Efficacy evaluation

Without knowing the results of the research data, the deputy chief physician of imaging and the deputy chief physician of radiotherapy evaluated the size, location, and lymph node metastasis of the tumors through esophagography and contrast-enhanced CT of the chest, abdomen, and neck. According to the Response Evaluation Criteria in Solid Tumors 1.1 (RECIST1.1), the efficacy evaluation results were divided into the following four levels. Complete response (CR) was defined as the disappearance of all lesions. Partial response (PR) was defined as *a* > 30% reduction in the longest diameter of the target lesion relative to baseline. Progressive disease (PD) was defined as the appearance of new target lesions or an increase in target lesions of <20%. Stable disease (SD) was defined as a reduction in lesion size by <30% or an increase in lesion size by <20%. Overall response rate (ORR) = CR + PR, non-objective response rate (NOR) = PD + SD, and incomplete response (IC)= PR + PD + SD

### 2.8. Data analysis

The flowchart was analyzed using FlowJo V10 software, and the data were processed using SPSS 25.0. Continuous variables (lesion length, PTV, and GTV) were converted into binary variables using the cutoff values calculated from the receiver operating characteristic (ROC) curve. The optimal cutoff point is the maximum point of the Jorden index, which is the sum of the sensitivity and specificity. The KS test (Kolmogorov–Smirnov test) was used for the positive analysis of each group of measurement data, and The LEVENE test was used to test the homogeneity of variance between the two groups. Normally distributed continuous variables are represented as mean and standard deviation (x ± s). A paired T test was used to compare paired data between the two groups before and after radiotherapy, and continuous variables that were not normally distributed are expressed as medians and interquartile ranges. The Mann-Whitney U test was used to compare the two groups that did not meet the normal distribution. The Wilcoxon signed-rank test was used for the paired data of the two groups before and after treatment. The two groups of data were analyzed for correlations. If they were consistent with a normal distribution, a Pearson’s correlation test was used; however, if they were not consistent, a Spearman’s correlation test was used. Statistical significance was set at a *P*-value of <0.05.

## 3. Results

### 3.1. Relationship between T lymphocyte subpopulations, NK cells, and PD-1 expression before CRT and clinicopathological characteristics

The relationship between T lymphocyte subpopulations, NK cells, and PD-1 expression before CRT and clinicopathological characteristics is shown in Table 1 and Table S2. There was no significant difference between the immunological parameters of patients with ESCC (*n* = 73) before CRT and age, sex, lesion location, N stage, and M stage (*p* > 0.05), while the lesion length and PD-1 expression in CD3^+^ and CD4^+^T cells was statistically significant (*p* < 0.05), and the GTV had a statistical difference with the ratio of CD4^+^/CD8^+^ and CD4^+^ T cells (*p* < 0.05).

**Table 1.** Correlation between the PD-1 expression in T-lymphocyte subsets and clinical pathological characteristics before chemoradiotherapy.

Characteristics	Cases	PD-1^+^CD3^+^%	*P* value	PD-1^+^CD4^+^%	*P* value	PD-1^+^CD8^+^%	*P* value
Age(years)			0.372		0.641		0.187
≤70	20	35.57(23.03)		35.84(27.85)		34.72(26.66)	
>70	53	31.58(18.86)		38.85(23.35)		29.84(22.32)	
Sex			0.208		0.760		0.136
Male	44	35.91 ± 12.50		41.23 ± 15.18		34.95 ± 13.26	
Female	29	31.89 ± 12.47		40.10 ± 13.33		28.64 ± 14.57	
Location			0.916		0.707		0.277
Upper	22	34.75 ± 11.72		43.15(19.00)		28.33 ± 14.21	
Middle	33	34.53 ± 13.14		39.25(28.64)		35.98 ± 14.43	
Lower	18	33.11 ± 10.55		40.77(21.54)		30.18 ± 12.30	
N-stage			0.128		0.315		0.390
N0	32	31.18 ± 10.01		38.49(18.69)		29.40 ± 12.95	
N1	27	38.82 ± 13.51		46.07(26.56)		35.20 ± 15.97	
N2-3	14	34.69 ± 12.81		39.20(29.23)		36.01 ± 11.58	
M-stage			0.354		0.552		0.804
M0	59	33.38 ± 12.67		40.18 ± 13.59		31.99 ± 14.31	
M1	14	36.97 ± 12.64		42.83 ± 15.06		33.09 ± 15.33	
Length			0.022		0.014		0.077
≤5	20	40.95 ± 14.41		49.07 ± 15.61		38.24 ± 13.12	
>5	53	32.25 ± 11.54		38.40 ± 13.11		30.51 ± 14.18	
GTV			0.271		0.549		0.502
≤34	27	32.57(21.67)		39.47 ± 15.56		30.09 ± 15.16	
>34	46	35.64(19.98)		41.96 ± 13.65		33.41 ± 13.36	

GTV, gross tumor volume.

### 3.2. Effects of CRT on T lymphocyte subsets and NK cells in patients with ESCC

**Figure 2.**
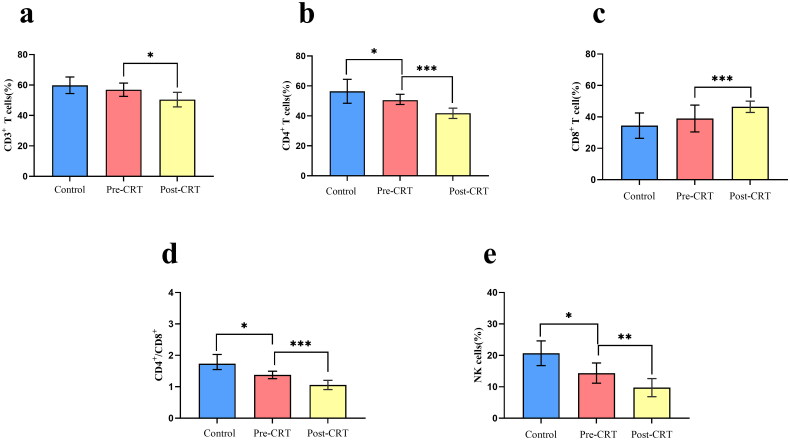
Comparison of NK cells and T lymphocyte subsets between patients with esophageal squamous cell carcinoma and healthy controls for. T test level, *p < 0.05, **p < 0.01, ***p < 0.001.

First, patients’ immune indicators were evaluated. The results are presented in Table 2 and Figure 2. The CD4^+^T cells, NK cells, and CD4^+^/CD8^+^ ratio in the peripheral blood of patients with ESCC before CRT were significantly lower than those in the control group (*n* = 10; all *p* < 0.05). Although CD8^+^ T cells were higher and CD3^+^ T cells were lower in patients with ESCC than in the healthy group, there were no statistically significant differences (*p* > 0.05). Compared with the Pre-CRT group, the CD3^+^ T cells (*p* = 0.018), CD4^+^ T cells (*p* < 0.001), NK cells (*p* = 0.009), and CD4^+^/CD8^+^ ratio (*p* < 0.001) in the peripheral blood of patients with ESCC after CRT were significantly reduced, and CD8^+^ T cells were significantly increased compared with those before CRT (*p* < 0.001).

**Table 2.** Comparison of NK cells and T lymphocyte subsets between patients with esophageal squamous cell carcinoma and healthy controls.

	Pre-CRT	Post-CRT	Control	Control: Pre-CRT	Post-CRT: Pre-CRT
Parameters	(*n* = 73)	(*n* = 73)	(*n* = 10)	*P* value	*P* value
CD3^+^%	57.57 ± 7.92	50.45 ± 8.65	59.86 ± 8.05	0.637	0.018
CD4^+^%	50.08 ± 7.90	41.49 ± 10.37	56.47 ± 11.18	0.031	<0.001
CD8^+^%	38.88 ± 8.84	46.47 ± 11.10	35.21 ± 9.73	0.237	<0.001
CD4^+^/CD8^+^	1.38 ± 0.47	1.06 ± 0.51	1.81 ± 0.55	0.023	<0.001
NK cells%	14.36 ± 5.82	9.75 ± 4.77	20.69 ± 5.50	0.020	0.009

CRT, chemoradiotherapy.

### 3.3. Effect of CRT on the expression of PD-1 in T lymphocyte subsets in patients with ESCC

**Figure 3.**
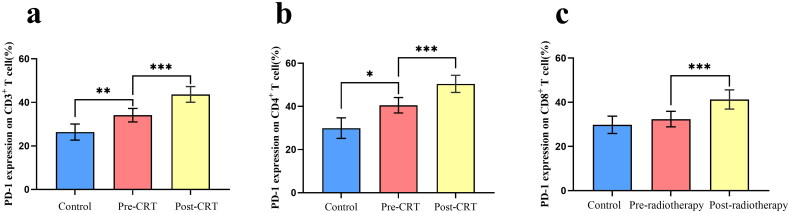
Comparison of PD-1 expression on T lymphocyte subsets between patients with esophageal squamous cell carcinoma and healthy controls. T test level, *p < 0.05, **p < 0.01, ***p < 0.001.

As shown in Table 3 and Figure 3, compared with normal controls, the expression of PD-1 in CD4^+^ and CD3^+^ T cells from patients with ESCC was significantly higher (all *p* < 0.05). However, the trend of increasing PD-1 expression in CD8^+^ T cells was weak and not statistically significant (*p* = 0.603). Compared with the Pre-CRT group, PD-1 expression in CD3^+^, CD4^+^, and CD8^+^ T cells of patients with ESCC increased significantly after CRT (all *p* < 0.001). The results showed that tumors increased the expression of PD-1 in T lymphocyte subsets and that CRT further increased the expression of PD-1. An increase in these indicators can be used to support the application of radiotherapy and chemotherapy in combination with PD-1 blockers.

**Table 3.** Comparison of PD-1 expression on T lymphocyte subsets between patients with esophageal squamous cell carcinoma and healthy controls.

	Pre-CRT	Post-CRT	Control	Control: Pre-CRT	Post-CRT: Pre-CRT
Parameters	(*n* = 73)	(*n* = 73)	(*n* = 10)	*P* value	*P* value
PD1^+^CD3^+^%	35.66 ± 12.79	43.65 ± 13.01	26.36 ± 5.15	0.007	<0.001
PD1^+^CD4^+^%	41.70 ± 14.66	50.44 ± 12.32	31.25 ± 7.60	0.015	<0.001
PD1^+^CD8^+^%	33.30 ± 14.12	41.11 ± 14.05	30.81 ± 5.48	0.603	<0.001

CRT, chemoradiotherapy.

### 3.4. Effect of chemotherapy on PD-1 expression in T lymphocyte subsets in patients with ESCC

Furthermore, we compared the differences in PD-1 expression in T lymphocyte subsets between the two groups of patients with ESCC after receiving definitive CRT and radiotherapy alone, as shown in Table 4. Compared with that in the radiotherapy alone group, the expression of PD-1 on CD3^+^ and CD4^+^ T cells in the CRT group showed little change (*p* > 0.05). However, in the CRT group, PD-1 expression on CD8^+^ T cells increased significantly (mean frequency 33.57 ± 11.95 vs. 43.27 ± 13.77, *p* < 0.05).

**Table 4.** Comparison of PD-1 expression on T lymphocyte subsets of esophageal squamous cell carcinoma between chemoradiation and radiotherapy groups.

Parameters	No chemotherapy *N* = 30	Chemotherapy *N* = 43	t value	*P* value
PD1^+^ CD3^+^%	39.39 ± 12.81	43.63 ± 10.99	0.970	0.337
PD1^+^ CD4^+^%	47.63 ± 14.78	47.19 ± 13.06	0.150	0.881
PD1^+^ CD8^+^%	33.57 ± 11.95	43.27 ± 13.77	2.074	0.043

### 3.5. Comparison of T lymphocyte subsets, NK cells, and PD-1 expression in different target areas in patients with ESCC after radiotherapy

Statistical analysis of immune indicators of ESCC on PTV shows that the minimum volume of the patient’s PTV is 106.20 cm^3^, the maximum volume is 892.60 cm^3^, the median volume is 262.80 cm^3^, and the average volume is 293.63 cm^3^ (Table 5). PTV, which was deemed a continuous variable, was converted into a binary variable using cut-off values calculated from ROC curves, with target volumes classified as ≥256.85 cm^3^ (*n* = 42) or <256.85 cm^3^ (*n* = 31). Our research results show that at the end of radiotherapy, the expression of PD-1 in CD3^+^, CD4^+^, and CD8^+^ T cells in the <256.85 cm^3^ group was reduced, and immune parameters such as CD3^+^T cells, CD4^+^ T cells, NK cells, and CD4^+^/CD8^+^ratio were significantly increased (all *p* < 0.05).

**Table 5.** Comparison of NK cells, PD-1 expression, and T lymphocyte subsets in different target volumes for patients with esophageal squamous cell carcinoma.

Parameters	PTV < 256.85 cm^3^	PTV ≥ 256.85 cm^3^	t value	*P* value
PD1^+^CD3^+^%	37.72 ± 12.57	46.85 ± 11.88	2.463	0.017
PD1^+^CD4^+^%	42.15 ± 14.28	51.26 ± 12.33	2.128	0.038
PD1^+^CD8^+^%	35.74 ± 14.50	45.73 ± 14.01	2.333	0.024
CD3^+^%	54.16 ± 12.42	50.49 ± 11.44	2.098	0.036
CD4^+^%	45.58 ± 9.72	38.45 ± 12.51	2.253	0.029
CD8^+^%	44.29 ± 11.52	48.66 ± 12.64	1.304	0.198
CD4^+^/CD8^+^	1.16 ± 0.66	0.83 ± 0.31	2.207	0.032
NK cells%	8.97 ± 4.39	7.80 ± 3.70	2.301	0.021

PTV, planning target volume.

### 3.6. Correlation of PD-1 expression in T lymphocyte subsets

**Figure 4.**
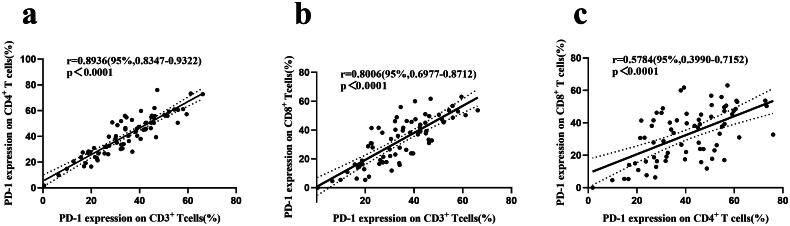
Correlation of PD-1 expression in T lymphocyte subsets. (a) PD-1 expression on CD3^+^ T cells vs. PD-1 expression on CD4^+^ T cells. (b) PD-1 expression on CD3^+^ T cells vs. PD-1 expression on CD8^+^ T cells. (c) PD-1 expression on CD4^+^ T cells vs. PD-1 expression on CD8^+^ T cells. r = Pearson correlation coefficient; *p* < 0.05, correlation analysis shows statistical significance.

To further explore the correlation between PD-1 expression levels in T lymphocyte subsets in patients with ESCC, we performed Pearson’s correlation analysis, and the results are shown in Figure 4. The results showed that PD-1^+^ CD3^+^ T cells were generally linearly correlated with PD-1^+^CD4^+^ T cells (*r* = 0.8936, *p* < 0.0001) and PD-1^+^ CD8^+^ T cells (*r* = 0.8006, *p* < 0.0001). This indicates that the higher the PD-1 expression of total T lymphocytes, the higher the proportion of PD-1 expression in CD4^+^ T cells and CD8^+^ T cells. In the correlation analysis between PD-1 expression in CD4^+^ and CD8^+^ T cells, it was concluded that there was a moderate positive correlation between PD-1^+^CD4^+^ and PD-1^+^CD8^+^ T cells (*r* = 0.5784, *p* < 0.0001).

### 3.7. Effects of PD-1 expression on short-term outcomes of T lymphocyte subsets in patients with ESCC before CRT

The short-term outcomes of CRT in patients, evaluated according to the RECIST criteria, are shown in Table 6. In this study, 53 cases (16 with CR and 37 with PR) were judged to have ORR, and 20 cases were judged to have NOR (19 with SD and 1 with PD). Among patients who achieved ORR, PD-1 expression on CD3^+^, CD4^+^, and CD8^+^ T cells was significantly reduced (all *p* < 0.05). In addition, we found a significant difference in PD-1 expression on CD8+ T cells between the CR and IC groups (mean frequency 29.94 ± 14.53 vs. 39.50 ± 14.42, *p* = 0.027). Therefore, our study shows that the expression of PD-1 in T lymphocyte subsets, especially CD8^+^ T cells, can predict the short-term outcomes of patients with ESCC and that patients with lower PD-1 expression in the peripheral blood have better short-term outcomes.

**Table 6.** Effect of PD-1 expression of T lymphocyte subsets on the short-term outcome of patients with esophageal squamous cell carcinoma.

	Cases *N* = 73(%)	CD3^+^PD-1^+^%	*P* value	CD4^+^PD-1^+^%	*P* value	CD8^+^PD-1^+^%	*P* value
Localized Objective Response							
ORR	53(72.60)	27.99 ± 10.14	0.037	34.80 ± 12.52	0.044	25.33 ± 13.29	0.020
IRR	20(27.40)	36.41 ± 14.51		42.97 ± 15.08		35.25 ± 14.45	
Localized Objective Response							
CR	16(21.92)	31.87 ± 13.74	0.078	38.18 ± 15.81	0.277	29.94 ± 14.53	0.027
IC	50(78.08)	39.50 ± 11.83		43.88 ± 13.42		39.50 ± 14.42	

ORR, objective response rate; NOR, non-objective response rate; CR, complete response; IC, incomplete response.

## 4. Discussion

EC is a prevalent malignant tumor that is often treated with definitive CRT in unresectable cases. The effectiveness of this treatment is influenced by numerous complex factors [13]. The combination of radiotherapy or CRT with immunotherapy has garnered increasing interest, prompting discussions on the optimal use of immunosuppressants in clinical practice. In this study, we aimed to investigate the correlation between immune markers, clinicopathological characteristics, and short-term outcomes in patients with ESCC and to lay the groundwork for the potential use of immunosuppressants.

The functional status of immune cells in the tumor microenvironment is essential for effective antitumor immunity. Maintenance of a normal immune status relies on the coordination of different immune cells, particularly peripheral blood T lymphocyte subsets [14]. The surface marker of T lymphocytes is CD3. T cells are categorized into CD4^+^ T cells (helper T cells) with a molecular phenotype of CD3^+^CD4^+^CD8^-^ and CD8^+^ T cells (cytotoxic T cells) with a molecular phenotype of CD3^+^CD4^-^CD8^+^. Helper T cells, or Th cells, can stimulate B lymphocyte differentiation and antibody production, as well as support cytotoxic T cells and macrophages in immune functions [15]. Cytotoxic T cells recognize infected cells by recognizing pathogenic antigens and subsequently kill them [16]. The CD4/CD8 ratio is a reflection of the immune balance within the body. A decrease in the CD4/CD8 ratio suggests immunosuppression and serves as a remarkable indicator of immune function in individuals with EC [17]. NK cells are cytotoxic effector cells of the innate immune system. They can identify and eradicate tumor cells without prior antigen exposure. NK cells are the body’s first line of defense for monitoring and killing tumors and play an important role in anti-tumor immunity and virus-infected cells [18]. In this study, the percentages of CD4^+^ T and NK cells and the CD4^+^/CD8^+^ ratio in the peripheral blood of patients with ESCC before CRT were considerably lower than those in the control group. This indicates the presence of immunosuppression, possibly due to the production or secretion of inhibitory soluble factors during tumor formation and development. These factors may deactivate immune effector cells, leading to a decrease in overall immune function.

Many studies showed that chemotherapy and radiotherapy have suppressive effects on the tumor immune environment in ESCC [19, 20]. We observed a marked decrease in CD3^+^ T cells, CD4^+^ T cells, NK cells, and the CD4^+^/CD8^+^ ratio after CRT, as shown in Table 2, and the percentage of CD8+ T cells increased slightly at the end of CRT. These findings indicate that CRT for ESCC may negatively affect the immune microenvironment. This can be explained by the fact that a combination of chemotherapy and radiotherapy can increase the sensitivity of malignant tumors to radiotherapy. Moreover, while radiation kills tumor cells, it also non-selectively kills T lymphocytes, resulting in low immune function and suppressed immune responses.

PD-1 is a member of the immunoglobulin superfamily. It is mainly expressed on the surface of CD4^+^ and CD8^+^ T cells and plays an important role in regulating tumor immune function [21]. Under the pathological conditions of cancer, abnormal PD-1 expression leads to immune cell dysfunction, inhibits T cell responses, and promotes tumor or infection progression through immunosuppression [22, 23]. Therefore, clarifying the expression level of PD-1 in peripheral blood is of great significance in detecting the immune function of patients with tumors and guiding the application of PD-1 inhibitors. Studies have shown that in studies related to gastric cancer, high expression of PD-1 is found in CD4^+^ and CD8^+^T cells [24, 25]. The results of this study showed that PD-1 expression on CD3^+^ and CD4^+^ T cells in the peripheral blood of patients with EC was considerably higher than that in the control group. The results of our study are similar to those reported previously. We also found that although the expression of PD-1 on CD8^+^ T cells in the EC group showed an upward trend compared to that in the control group, the difference was not statistically significant. However, when we performed a correlation analysis on the expression levels of PD-1 on CD4^+^ and CD8^+^ T cells, the two were markedly positively correlated; therefore, PD-1 expression may have positive effects on CD8^+^ T cells when the number of cases increases. Accordingly, high expression of PD-1 in the peripheral blood of tumor patients may inhibit the activation and proliferation of T lymphocytes and weaken the antitumor immune effect of T lymphocytes, forming an immune-tolerant tumor microenvironment. The above results suggest that detecting PD-1 expression in the peripheral blood is expected to become a clinical indicator for monitoring the immune function of patients with EC and guiding the application of PD-1 immunosuppressants.

Evidence for the direct effect of radiation on PD-1 and ligand upregulation was previously observed in mouse models of breast cancer and melanoma, where radiation upregulated PD-1 expression in CD8^+^ T cells [26, 27]. Da Silva, et al. [28] reported that increased PD-1 expression in T lymphocyte subsets in patients with cervical cancer after CRT. The analysis of this study showed that compared to those before CRT, PD-1 expression levels in CD3^+^, CD4^+^, and CD8^+^ T cells were significantly increased after CRT (*p* < 0.001). This shows that CRT can increase the expression of PD-1 in T lymphocyte subpopulations. Wei, et al. [29] has studied increased PD-1 expression in T lymphocyte subsets in patients with ESCC after CRT. Similarly, in this study, we found that in patients with ESCC, the expression of PD-1 on CD8^+^ T cells was considerably higher in the definitive CRT group than in the radiotherapy alone group. This suggests that chemotherapy increases PD-1 expression in CD8+ T cells. The marked increase in PD-1 expression after radiotherapy and chemotherapy strongly suggested radiotherapy- and chemotherapy-induced immune suppression. Several clinically available antibodies block PD-1 signaling (e.g. nivolumab, pembrolizumab, and lambrolizumab). Moreover, multiple preclinical studies using mouse models have shown that gene deletion of PD-1 in mice considerably improves the systemic antitumor efficacy of radiotherapy [30, 31]. In addition, radiotherapy in immunotherapy can not only enhance the anti-tumor immune response but also prevent tumor cells from becoming resistant to drugs [32, 33]. Therefore, we speculated that combining PD-1 immunosuppressants with radiotherapy could improve the efficacy and reduce drug resistance. The optimal time for PD-1 immunosuppressive administration may be at the end of CRT.

In addition to the relative enhancement of non-specific immunity, both cellular and humoral immunity are strongly suppressed by radiotherapy in patients with EC [34]. We further found that the size of the radiotherapy target volume also had an impact on the body’s immune status. This may be because a larger radiotherapy target volume correspondingly increases the damaging effect of radiation on immune cells, thus aggravating immune suppression. In this study, patients with a larger PTV had significantly lower CD3^+^ T cells, CD4^+^ T cells, NK cells, and CD4^+^/CD8^+^ ratios (*p* < 0.05), which further supports the overall immunosuppressive effect of radiotherapy on the systemic immune cell environment in patients with EC. In addition, in this study, the expression of PD-1 on CD4^+^, CD8^+^, and CD3^+^ T cells was significantly increased compared to that in the PTV < 256.85 cm^3^ group (*p* < 0.05), which once again demonstrated that radiotherapy can further increase the expression of PD-1 in peripheral blood to a certain extent. Therefore, radiotherapy combined with PD-1 immunosuppressants may be beneficial.

High PD-1 expression negatively regulates T cell activation and proliferation, leading to T cell functional exhaustion and tumor growth. Some studies have shown that PD-1 is overexpressed in T lymphocyte subsets in the peripheral blood of various malignant tumors, such as lung, breast, and gastric cancers [35, 36]. Some researchers found that PD-1 expression in T lymphocyte subsets is related to the overall survival rate of patients with ESCC [37]. In addition, Zheng et al. [38] found that high PD-1 expression in CD4^+^ T cells in the circulating blood of patients with non-small cell lung cancer was associated with shorter progression-free and overall survival. Shen et al. [39] found that PD-1 expression on CD8^+^ T cells in pancreatic ductal adenocarcinoma was related to the overall survival rate and clinicopathological characteristics, such as clinical stage. These results suggest that PD-1 is a potential prognostic marker. In this study, we further analyzed the impact of PD-1 expression in T lymphocyte subpopulations on short-term efficacy in patients. PD-1 expression in CD3^+^, CD4^+^, and CD8^+^ T cells was significantly reduced in patients who achieved ORR (all *p* < 0.05). Compared with the IC group, the number of PD-1^+^CD8^+^ T cells was significantly reduced in the CR group (*p* < 0.05). The results showed that patients with high PD-1 expression in T lymphocyte subsets, especially CD8^+^ T cells, had a considerably lower ORR or CR. This suggests that PD-1 in the circulating blood may be a potential indicator for judging the short-term outcome of ESCC. Therefore, clarifying the expression level of PD-1 in the peripheral blood is of great significance in predicting the short-term outcome of ESCC and guiding the application of PD-1 inhibitors.

Our study had some limitations. One major limitation is that while PD-1 and other immune indicators have been found to be more highly expressed in tumor infiltration compared to circulating T cells [40], it is challenging to measure the level of PD-L1 expression in tumor tissue during definitive radiotherapy. Therefore, our analysis was limited to the peripheral blood T cells. Although many immune processes are expected to be similarly regulated in both tumor tissue and circulating immunity, we believe that our findings would have been more precise if we had sampled the tumor tissue. Another limitation is the relatively small sample size and the limited number of healthy controls in our study, resulting in some of our study’s *p*-values not reaching statistical significance. Future studies should aim to increase the sample size to obtain more robust results.

## 5. Conclusions

In our study, CRT further reduced the overall immune function of patients with EC, and the degree of decline was related to the irradiation area. Therefore, enhancing the immune function during CRT may positively affect patient prognosis. In this study, during dynamic monitoring of immune function, we found that patients with ESCC had higher expression of PD-1 in peripheral blood cells than that in healthy individuals. In particular, after radiotherapy and chemotherapy, PD-1 expression in T lymphocytes increased considerably, suggesting that the sequential application of PD-1 immunosuppressants after radiotherapy and chemotherapy can be beneficial. Moreover, a correlation was observed between PD-1 expression prior to CRT and short-term outcomes of patients with EC. Thus, PD-1 expression on T lymphocytes may serve as a potential indicator for predicting the short-term outcomes of ESCC.

## Supplementary Material

Supplementary Material.docx

## Data Availability

The raw data supporting the conclusions of this article will be made available by the authors without undue reservation.
