# Supplementary material for: Changes in PD-1 expression on T lymphocyte subsets and related immune indicators before and after definitive chemoradiotherapy for esophageal squamous cell carcinoma
Source: Ann Med. 2024 Dec 23;57(1):2445190. doi: 10.1080/07853890.2024.2445190 (PMC11703528; doi:10.1080/07853890.2024.2445190)
Supplement: Supplementary Material.docx [file IANN_A_2445190_SM3811.docx]

Table S 1 Patients, tumors, and treatments characteristics

| Characteristic | No. of patient (N=73) | % |
| --- | --- | --- |
| Age |  |  |
| ≤70 | 20 | 27.40 |
| ＞70 | 53 | 72.60 |
| Sex |  |  |
| Male | 44 | 60.27 |
| Female | 29 | 39.73 |
| Smoking |  |  |
| Former or current | 30 | 41.10 |
| Never | 43 | 58.90 |
| Drinking |  |  |
| Former or current | 32 | 43.84 |
| Never | 41 | 56.16 |
| Location |  |  |
| Upper | 22 | 30.14 |
| Middle | 33 | 45.21 |
| Lower | 18 | 24.65 |
| N-stage |  |  |
| N0 | 32 | 43.83 |
| N1 | 27 | 36.99 |
| N2-3 | 14 | 19.18 |
| M-stage |  |  |
| M0 | 59 | 80.82 |
| M1 | 14 | 19.18 |
| Length |  |  |
| ≤5 | 20 | 27.40 |
| ＞5 | 53 | 72.60 |
| Treatment modality |  |  |
| RT alone | 28 | 38.36 |
| CCRT | 45 | 61.64 |
| GTV |  |  |
| ≤34 | 27 | 36.98 |
| ＞34 | 46 | 60.01 |

RT, radiotherapy; CRT, chemoradiotherapy; GTV, gross tumor volume.

Table S 2 The correlation between the T lymphocyte subpopulations and clinical pathological characteristics before radiotherapy

| Clinical pathological characteristics | Cases | CD3^+^% | P Value | CD4^+^% | P Value | CD8^+^% | P Value | CD4^+^/CD8^+^ | P Value | NK cells% | P value |
| --- | --- | --- | --- | --- | --- | --- | --- | --- | --- | --- | --- |
| Age(years) |  |  | 0.604 |  | 0.166 |  | 0.273 |  | 0.178 |  | 0.462 |
| ≤70 | 20 | 57.55±8.28 |  | 52.11±6.79 |  | 37.07±7.87 |  | 1.49±0.43 |  | 12.25±4.58 |  |
| ＞70 | 53 | 58.72±9.64 |  | 49.06±7.88 |  | 39.81±8.83 |  | 1.31±0.46 |  | 12.87±5.49 |  |
| Sex |  |  | 0.250 |  | 0.452 |  | 0.587 |  | 0.623 |  | 0.441 |
| Male | 44 | 56.17±8.96 |  | 51.35±8.45 |  | 39.27±9.57 |  | 1.40±0.51 |  | 13.86±4.41 |  |
| Female | 29 | 52.12±6.64 |  | 49.85±6.51 |  | 38.09±7.16 |  | 1.34±0.38 |  | 11.21±4.45 |  |
| Location |  |  | 0.225 |  | 0.374 |  | 0.240 |  | 0.720 |  | 0.492 |
| Upper | 22 | 55.28±9.41 |  | 47.23±10.61 |  | 35.92±8.48 |  | 1.45±0.45 |  | 11.98±4.88 |  |
| Middle | 33 | 59.05±11.03 |  | 51.02±7.83 |  | 40.56±8.34 |  | 1.34±0.41 |  | 14.11±4.35 |  |
| Lower | 18 | 58.54±10.03 |  | 48.66±9.47 |  | 38.73±9.14 |  | 1.36±0.55 |  | 11.48±4.15 |  |
| N-stage |  |  | 0.867 |  | 0.789 |  | 0.896 |  | 0.960 |  | 0.695 |
| N0 | 32 | 58.02±9.40 |  | 49.54±7.62 |  | 38.19±8.41 |  | 1.32±0.59 |  | 13.94±5.43 |  |
| N1 | 27 | 58.75±8.59 |  | 50.27±8.60 |  | 39.02±10.05 |  | 1.42±0.81 |  | 13.23±5.98 |  |
| N2-3 | 14 | 57.70±8.66 |  | 51.60±6.95 |  | 39.46±6.84 |  | 1.48±0.65 |  | 10.54±6.65 |  |
| M-stage |  |  | 0.124 |  | 0.090 |  | 0.048 |  | 0.070 |  | 0.485 |
| M0 | 59 | 50.55 ± 8.96 |  | 49.25±7.88 |  | 39.95±8.51 |  | 1.31±0.44 |  | 14.52±4.39 |  |
| M1 | 14 | 55.72 ± 8.64 |  | 53.39±6.98 |  | 35.55±7.87 |  | 1.57±0.43 |  | 13.10±5.56 |  |
| Length |  |  | 0.367 |  | 0.376 |  | 0.132 |  | 0.833 |  | 0.419 |
| ≤5 | 20 | 58.10±8.76 |  | 48.02±9.33 |  | 35.50±7.78 |  | 1.33±0.36 |  | 11.32±4.66 |  |
| >5 | 53 | 62.04±10.10 |  | 50.32±7.63 |  | 39.65±8.88 |  | 1.37±0.48 |  | 12.28±4.53 |  |
| GTV |  |  | 0.263 |  | 0.031 |  | 0.153 |  | 0.049 |  | 0.238 |
| ≤34 | 27 | 54.65±10.55 |  | 47.26±8.65 |  | 40.49±9.34 |  | 1.22±0.41 |  | 10.15±5.65 |  |
| >34 | 46 | 58.40±12.27 |  | 52.05±6.74 |  | 37.23±8.06 |  | 1.48±0.46 |  | 14.98±7.65 |  |

GTV, gross tumor volume
